# Healthcare Chaplaincy for Geriatric Patients: A Quasi-Experimental Study into the Outcomes of Catholic Chaplaincy Interventions in Belgium

**DOI:** 10.1007/s10943-023-01982-6

**Published:** 2024-01-19

**Authors:** Lindsy Desmet, Jessie Dezutter, Anne Vandenhoeck, Annemie Dillen

**Affiliations:** 1 Faculty of Theology and Religious Studies, KU Leuven, Sint-Michielsstraat 6, 3000 Leuven, Belgium; 2 Faculty of Psychology and Educational Sciences, KU Leuven, Leuven, Belgium

**Keywords:** Outcome, Chaplaincy, Geriatric care, Spiritual care, Healthcare

## Abstract

The present non-randomized clinical trial examined the short-term outcomes of one-on-one chaplaincy interventions with 416 geriatric patients in Belgium. Participants were interviewed one or two days before a potential chaplaincy intervention (baseline measurement), and one or two days after a potential intervention (post-measurement). Patients in the non-randomized intervention group received an intervention by the chaplain, while the non-randomized comparison group did not. Patients in the intervention group showed a significant decrease in state anxiety and negative affect, and a significant improvement in levels of hope, positive affect, peace, and Scottish PROM-scores, compared to the comparison group. Levels of meaning in life and faith did not significantly change after the chaplaincy intervention. This study suggests that geriatric patients may benefit from chaplaincy care and recommends the integration of chaplaincy care into the care for older adults.

## Introduction

Aging is a complex process in which the spirituality of older persons evolves and can become more prominent (Moberg, 2001). Stressful and life-changing situations in late life such as the loss of loved ones, the decline of physical and cognitive abilities, and hospitalization can trigger the spiritual dimension of people. On the one hand, people’s spirituality can be a powerful resource to cope with these stressful events (Park, 2007). On the other hand, stressful events can provoke intensified spiritual needs and spiritual distress (Koenig et al., 1995; Moberg, 2005; Wink & Dillon, 2002). For example, doubts can occur about one’s meaning in life, dignity, roles in their (past) life, and about the trust in themselves, others, the world, and/or the transcendent (MacKinlay, 2006).

During hospitalization, spiritual needs and spiritual distress can be addressed by healthcare professionals, especially chaplains. Hospital chaplains provide professional spiritual care and are part of an interdisciplinary healthcare team. They can support older adults in the search for meaning in life, reconciliation with (past) life, experiencing peace and hope, coping with death, and in reflecting on and deepening the role of spirituality in one’s life (Prause et al., 2020; Timmins et al., 2018; Visser et al., 2023; Wells et al., 2021). Recent research has shown that patients feel satisfied when they receive chaplaincy care and that their spiritual needs are met (Kirchoff et al., 2021; Marin et al., 2015; Muehlhausen et al., 2022; Tan et al., 2020). Also, patients highly appreciate the chaplain's presence, trusting relationship, attentive listening, and familiarity (McCormick & Hildebrand, 2015; Sailus, 2017).

Of particular interest is what the impact is of healthcare chaplaincy on patients. Recently, several case studies have been published worldwide, providing crucial insights into the impact and outcomes of chaplaincy care (Fitchett & Nolan, 2015, 2018; Kruizinga et al., 2020). The downside is that they are written down from the chaplain’s perspective rather than the patient’s perspective and that they are limited to one individual case. To examine the impact across multiple patients or groups, more quantitative outcome-oriented research is needed (Handzo et al., 2014; Kelly & Vandenhoeck, 2017). The most rigorous method to do this is by using a randomized control trial (RCT). However, in healthcare chaplaincy studies, RCTs are scarce (Bay et al., 2008; Iler et al., 2001; Kruizinga et al., 2019).

Moreover, studies examining the outcomes of chaplaincy interventions generally suffer from four methodological limitations (see also Buelens et al., 2023; Jankowski et al., 2011). First, a comparison group is often lacking (Kestenbaum et al., 2017; Kevern & Hill, 2015). Because there is no comparison with a group that received no/an alternative intervention, it is difficult to know whether the effects in the intervention group are caused by the intervention of the chaplain. Second, results are frequently based on small sample sizes (Kestenbaum et al., 2017). Third, there are a large number of studies investigating spiritual care interventions provided by the whole multidisciplinary team (Piderman et al., 2014; Rabow et al., 2004; Sun et al., 2016). This makes it difficult to pinpoint the specific contribution of the chaplain. Fourth, studies investigating pre-defined intervention programs by the chaplain are interesting but do not give insights into the impact of healthcare chaplaincy in a real-life context, where chaplains usually offer unstructured interventions (Liefbroer et al., 2021).

In geriatric healthcare, two outcome studies on chaplaincy interventions have been carried out and one study design has been published. First, Baker’s (2001) study with independent-living, assisted-living, and residents with a need for nursing care or treatment in Pennsylvania, investigated the impact of chaplaincy interventions on depression. Participants were first matched according to their age, gender, and level of care, and then, one participant per match was assigned to the intervention group and one to the control group. The intervention group received weekly chaplaincy interventions for six months, while the control group received minimal chaplaincy interventions. Depression scores decreased in the intervention group after six months of interventions (post-measurement). Three months after the last intervention (follow-up measurement), depression scores increased in the intervention group. In the control group, depression scores increased both at post- and follow-up measurement. Second, Zhang and colleagues (2020) carried out a retrospective study with older adults in a rehabilitation unit of a long-term care facility in Boston and Dedham. The intervention group was visited by the chaplain; the control group was not. Compared to the control group, no significant changes in outcomes in the intervention group were found for mood, pain level, functional ability and discharge status, at three months and six months after the intervention. Third, an RCT-design has been published (Kittelson et al., 2019). The study aims to investigate the impact of dignity therapy provided by nurses or chaplains on older palliative care outpatients suffering from cancer. Outcomes that will be examined are dignity impact, preparation for death, life completion, and cancer prognosis awareness (Kittelson et al., 2019).

In short, Baker’s (2001) study found a diminished level of depression in the intervention group after receiving chaplaincy interventions, while Zhang’s (2020) study found no effect on mood, pain level, functional ability, and discharge status of rehabilitating older adults after receiving chaplaincy care. These initial findings need to be further investigated, along with other outcomes, and especially in older people with care needs. To gain more insight into the impact of healthcare chaplaincy on geriatric patients, the present study was conducted.

## Outcome Measures

The outcome measures tested in this study were selected based on the goals of healthcare chaplaincy and based on what chaplains do (Massey et al., 2015; Sharma et al., 2021). On the one hand, chaplains specifically address the spiritual needs and spiritual well-being of patients. On the other hand, chaplains also care for other aspects of patients’ well-being and distress, especially aspects of emotional and psychological well-being and distress (Visser et al., 2023). Therefore, we tested the impact of a chaplaincy intervention on spiritual outcome measures as well as psychological and emotional outcome measures. Since we are measuring short-term effects, we also selected outcomes that are able to fluctuate in time such as positive and negative affect.

For each outcome tested in this study, we shortly describe to what extent the outcome has been examined in current research on healthcare chaplaincy in different hospital populations. More information about the outcomes and how they are measured in this study can be found in the section “Measurements”.

*Anxiety.* Anxiety refers to the condition of feeling tense, restless, worried, and scared. In the small field of research on healthcare chaplaincy outcomes, anxiety is one of the most frequently examined outcomes. Three studies showed a decrease in anxiety in patients and family after receiving a visit/visits by the chaplain (Berning et al., 2016; Iler et al., 2001; Torke et al., 2023). Three studies found no effect of spiritual care interventions provided by volunteers or by chaplains on patients' anxiety (Bay et al., 2008; Buelens et al., 2023; Miles et al., 2021).

*Hope.* Hope is considered a dynamic, universal, and multidimensional life force that can be a powerful mechanism during illness (Dufault & Martocchio, 1985; Herth, 2000). Reflections on chaplaincy care state that chaplains are a hopeful presence in the encounter with the patient and that they identify patients’ hope (Nolan, 2011; VandeCreek & Lucas, 2001). Surprisingly, research on hope as an outcome of healthcare chaplaincy is very rare. A study with coronary artery bypass graft patients showed no significant change in the experience of hope after chaplaincy interventions (Bay et al., 2008).

*Positive and negative affect.* Positive affect refers to a positive mood such as feeling happy or excited, while negative affect refers to a negative mood such as feeling guilty, ashamed, or sad (Watson et al., 1988)*.* It has not yet been tested if patients’ mood or affect in general changes after a chaplaincy intervention. In psychotherapy, studies show an increase of positive affect and decrease of negative affect in depressed adults after psychotherapy interventions (Boumparis et al., 2016). Since chaplains offer emotional support and encourage patients to share their feelings, we assume that chaplaincy interventions may also have an impact on positive and negative affect (Sharma et al., 2021).

*Peace, meaning in life, and faith.* These three outcomes are discussed together here, because they are measured by the same instrument (see further). We consider each of the three outcomes as a distinct outcome. The focus on peace in this study is on the inner experience of peace, often called “inner peace” (Büssing & Koenig, 2010). Meaning in life is about experiencing life as significant, purposeful, and valuable (Dezutter et al., 2013). Faith indicates the role of religion/spirituality in people’s life. Studies showed an increase in patients’ peace/peacefulness after receiving chaplaincy interventions (Buelens et al., 2023; Piderman et al., 2017). The same was found in studies with spiritual care interventions by a multidisciplinary/interdisciplinary care team, including the chaplain (Piderman et al., 2014; Sun et al., 2016). Less is known about the impact chaplains have on patients’ meaning in life and faith. Of interest is the study by Kestenbaum and colleagues (2017) which showed an increase in faith after receiving chaplain visits and no change for peace and meaning in life.

*Scottish PROM*. Because we want to focus on outcomes that are closely related to what chaplains do, the work by Snowden and Telfer (2017) is of interest. They were the first to develop a validated instrument specifically designed to measure the outcomes of chaplaincy interventions, called “The Scottish Patient Reported Outcome Measure (PROM)” (Snowden et al., 2022b). The scale items will be further described below. A recent study in primary care demonstrated that when patients receive an intervention from the chaplain, they score higher on the PROM-scores, compared to their initial scores (Snowden et al., 2022a). However, this study lacked a comparison group. The same results were found in a study with inpatients on the outcomes of spiritual care interventions by a multidisciplinary team. This study did compare the outcome scores with a group of patients who did not receive spiritual care (Tan et al., 2020). However, the spiritual care in this study was provided by various caregivers, making it difficult to indicate the specific impact of the chaplain on the PROM-scores.

## Aim of the Study

This study aims to test the short-term effects of a single one-on-one hospital chaplaincy intervention on aspects of geriatric patients’ well-being and distress. The intervention was provided by lay healthcare chaplains, who are affiliated with the Roman Catholic Church. The outcomes tested in this study are the feelings of state anxiety, the experience of hope, positive and negative affect, peace, meaning in life, and faith. Besides these outcomes, the Scottish Patient Reported Outcome Measure (PROM) was included (Snowden & Telfer, 2017). We hypothesized that when patients receive an intervention by the chaplain, the outcome scores will significantly change compared to patients who receive no intervention by the chaplain. This while taking into account the baseline outcome scores of both groups and controlling for background variables. The preregistration of the study, published on the Open Science Framework website, offers a more detailed description of the hypotheses (osf.io/3r7em).

## Materials and Methods

### Study Design and Procedure

This study is a quasi-experimental study with a pretest–posttest nonequivalent comparison-group design in which geriatric patients were not randomly assigned to the intervention or comparison group. In 2022, hospitalized geriatric patients were selected on geriatric wards of three Roman Catholic hospitals in Flanders, Belgium. The chaplain, together with the head nurse, selected the patients eligible for this study. Patients eligible for inclusion in this study had to meet the following criteria: aged 65 years and over, capable of understanding and answering the questionnaires, able to express informed consent, and remaining hospitalized for a couple of days after the baseline measurement. Patients were excluded if they suffered from severe forms of memory loss or mental health problems or did not understand Dutch.

Participants were assigned to one of the two groups, based on whether or not they received a chaplaincy intervention. Participants were eligible for a chaplaincy intervention if they were referred to the chaplain, as described in the following section. If they received a chaplaincy intervention, they were assigned to the intervention group. Participants who were not referred to the chaplain, did not receive a chaplaincy intervention and were assigned to the comparison group. Patients in the comparison group also did not receive an alternative intervention. If a participant was referred to the chaplain, but the chaplain failed to visit the patient within the time frame, the patient was assigned to the comparison group. Each time the intervention and/or comparison group is mentioned in this study, we refer to the non-randomized intervention and non-randomized comparison group.

Participants were interviewed one or two days before a potential chaplaincy intervention (baseline measurement), and one or two days after a potential intervention (post-measurement). For example, on Monday, the baseline measurement took place for all study participants. On Tuesday, participants who were referred to the chaplain could receive a chaplaincy visit. Participants who were not referred to the chaplain, did not receive a chaplaincy intervention. On Wednesday, both intervention and comparison group participants participated in the post-measurement. Depending on the availability of interviewers, patients and chaplains, the timing may vary by one day. Both questionnaires at baseline and post-measurement were conducted orally by the first author and two master students in the hospital room of the patient. A power analysis (G*power, *d* = 0.20, α = 0.05) stated to include at least 123 participants in each group.

**Fig. 1:**
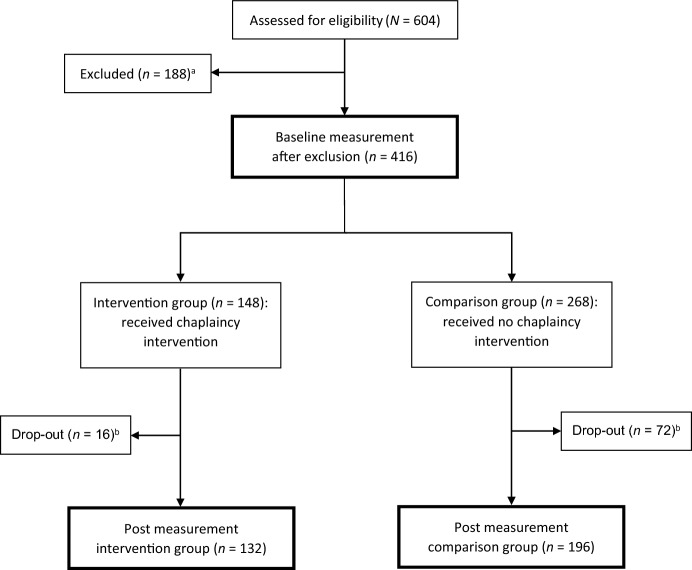
Flowchart of participants. *Note* ^a^Geriatric patients were not included in the study when they refused to participate, were hard of hearing, were too tired/sick, were not in their room, had visitors, were not considered capable enough by the interviewer, were infected with coronavirus, said they would stay in the hospital for less than three days, were under the age of 65, or had a psychiatric disorder. Other reasons for exclusion were technical problems and double participation in the study. ^b^ Patients dropped out of the study between the baseline and post-measurement, because of discharge from the hospital (*n* = 43), declining to participate (*n* = 14), being too ill or too tired (*n* = 12), being absent or transferred to another ward/hospital (*n* = 8), being too confused, nervous, or disabled to answer (*n* = 4), having visitors (*n* = 1), or because of the limited time of the interviewer (*n* = 6)

Figure 1 visualizes the flow of the data collection through the study. The study was approved by the Ethics Committee Research UZ/KU Leuven (S65939) and the local Ethics Review Board of each hospital.

### Referral to the Chaplain

Participants were referred to the chaplain when the referrer felt there was a need for chaplaincy care, based on his or her personal assessment of the presence of spiritual needs in the patient. When the referrer felt no need for chaplaincy care, the patient was not referred to the chaplain. There was no standardized protocol for referring to the chaplain and a referral could be made by different people, as this is usually the case in Belgian hospitals.

First, the referral could be made by any member of the healthcare team. Members of the healthcare team are familiar with chaplaincy care and are used to refer to the chaplain. They received no specific training on how and when to refer to the chaplain as part of this study.

Second, the referral could be made by the interviewer when a need for chaplaincy care or intense spiritual needs were observed during the baseline measurement. As with the referral by someone of the healthcare team, the interviewer's referral was based on a personal assessment.

Third, the patient, relatives or family of the patient, or someone else could ask for chaplaincy care for a patient via the healthcare team or directly to the chaplain.

Fourth, the chaplain could visit a patient on his/her own initiative. For example, based on an identified need for chaplaincy care during previous encounters.

### Study Sample

Participants were aged between 65 and 100 years, with a mean age of 82.53 years (*SD* = 6.40) in the comparison group and 82.30 years (*SD* = 6.11) in the intervention group. Except for five participants with Dutch nationality, all participants have Belgian nationality. Participants were admitted to the hospital for a couple of days (42.3%), one week (23.1%), two weeks (14.2%), or more than two weeks (20.2%). One answer was missing because the patient could not remember the length of stay in the hospital. Between the baseline and post-measurement, 17.4% of the total sample at post-measurement (*n* = 328) talked to someone other than the chaplain (such as their doctor, healthcare team, patient support team, family or loved ones, or roommate) about their doubts, worries, or fears. Three hundred and sixteen of the 416 participants identified themselves as Catholic. Other characteristics of the participants are shown in Table 1.

**Table 1:** Participant demographic characteristics

Baseline characteristic	Intervention group		Comparison group		Full sample	
	*n*	%	*n*	%	*n*	%
*Gender*						
Female	100	67.6	166	61.9	266	63.9
Male	48	32.4	102	38.1	150	36.1
*Civil status*						
Single	4	2.7	10	3.7	14	3.4
Married	57	38.5	107	39.9	164	39.4
Divorced	7	4.7	10	3.7	17	4.1
Widowed	78	52.7	135	50.4	213	51.2
Relationship/living together	2	1.4	6	2.2	8	1.9
*Highest educational level*						
Primary school^a^	17	11.5	16	6.0	33	7.9
Lower sec. education (< 16y)	69	46.6	137	51.1	206	49.5
Higher sec. education (< 18y)	45	30.4	82	30.6	127	30.5
University or college	17	11.5	33	12.3	50	12.0
*Importance of religion/spirituality in life*						
Not important	45	30.4	78	29.1	123	29.6
Somewhat important	14	9.5	37	13.8	51	12.3
Important	29	19.6	74	27.6	103	24.8
Very important	60	40.5	79	29.5	139	33.4
*Religious affiliation*						
Nonbeliever	33	22.3	50	18.7	83	20.0
Believer without religious affiliation	2	1.4	8	3.0	10	2.4
Believer with religious affiliation	113	76.4	210	78.4	323	77.6
*Religious identification*						
Christian^b^	110	74.3	208	77.6	318	76.4
Others^c^	4	2.7	11	4.1	15	3.6
Not applicable	34	23.0	49	18.3	83	20.0
*Praying (alone or with other people)*						
Never	48	32.4	101	37.7	149	35.8
Seldom	6	4.1	16	6.0	22	5.3
Occasionally	20	13.5	43	16.0	63	15.1
Weekly	10	6.8	17	6.3	27	6.5
Daily	64	43.2	91	34.0	155	37.3
*Religious activity (in group)*						
Never	49	33.1	108	40.3	157	37.7
Seldom	16	10.8	41	15.3	57	13.7
Occasionally	28	18.9	49	18.3	77	18.5
Weekly^d^	55	37.2	70	26.1	125	30.0

^a^Two participants stated that they did not go to school (one participant in the intervention group and one in the comparison group). In the analyses, we added these participants to this category to avoid categories that are too small

^b^All participants stated that they were Catholic, except for one person identifying as Protestant and one person identifying as Christian

^c^This category includes Secular Humanists (*n* = 3), Jehovah’s Witnesses (*n* = 3), Evangelicals (*n* = 1), Buddhists (*n* = 1), and people feeling connected with an ethical framework, the spiritual mystery, the nature, the self, or a philosophy (*n* = 7)

^d^One person in the comparison group is involved in daily religious activities

### The Chaplaincy Intervention

In total, 148 patients received a non-standardized intervention. Five interventions were not registered by the chaplains due to technical problems. All chaplains self-identified as Roman Catholic and were trained lay people who provide professional spiritual care adapted to the spiritual needs of the patients. Six female and one male chaplain were involved. They are employed by the hospitals and paid with subsidies the hospital receives from the government. The chaplains take care of every patient with spiritual needs regardless of their (non-)affiliation with religion/spirituality. Although one chaplain has one year of experience in healthcare chaplaincy, the other six chaplains each have at least seven years of experience. Patients received an intervention by the chaplain because they were referred to the chaplain by the interviewer (*n* = 74) or caregiver (*n* = 45), or because patients asked for it (*n* = 4), family asked for it (*n* = 2), or the chaplain took the initiative (*n* = 17). One encounter was by chance (*n* = 1). The majority of the interventions (*n* = 123) were a first encounter with the patient.

The chaplaincy interventions were individual, non-standardized conversations with the patients for at least 15 min. Each intervention was tailored to the individual needs of the patient. To assure the ecological validity of this study, i.e., to make sure that the results are not based on an artificial setting that does not take place in a real-life context, the conversations did not follow a fixed pattern.

For each intervention, the chaplains registered what they did. Examples of interventions that took place in this study are acknowledging patients’ feelings and current situation, helping them reflect on their life story, helping them find helpful resources to cope with their illness, and helping them deepen their personal spiritual/religious framework.

### Measurements

In order to grasp fluctuations in outcomes in a short time, patients were asked about the current presence of the outcomes.

*Feelings of state anxiety*. The term “state” refers to the temporal and situational condition of an emotion, feeling, or experience related to events in people’s life (Snyder et al., 1996; Spielberger & Reheiser, 2009). This means that the focus of the term “state” is on the transitory condition, not on someone’s personality traits. The state condition of anxiety was measured with the six-item version of the State Anxiety Inventory (SAI) (Marteau & Bekker, 1992). The scale includes items such as “I am tense” and “I am worried”. The Dutch translation was used with items scored on a 4-point Likert scale from *not at all* to *very much* (Van der Ploeg et al., 2000). At baseline and post-measurement, Cronbach’s α was, respectively, .80 and .81.

*Experience of hope*. Hope was measured by the Dutch version of the Herth Hope Index (HHI) (Herth, 1991; Van Gestel-Timmermans et al., 2010). The multidimensional scale comprises 12 items with answers rated on a 4-point Likert scale from *strongly disagree* to *strongly agree* (e.g., “I can see possibilities in the midst of difficulties”). Haugan and colleagues (2013) demonstrated that the HHI is a reliable and valid instrument to assess hope in nursing home patients. In this sample with geriatric patients, Cronbach’s α was .73 at baseline and .77 at post-measurement.

*Positive and negative affect*. The Positive and Negative Affect Scale (PANAS) determines the level of positive and negative mood in a person (Watson et al., 1988). Both types of affects are measured separately with ten items. Positive affect includes items such as feeling strong, feeling enthusiastic, and feeling proud. Negative affect deals with feelings of guilt, shame, and nervousness. Answer categories ranged on a 5-point Likert scale from *not at all* to *extremely*. The PANAS is considered a reliable instrument that is sensitive to changes over time (Magyar-Moe, 2009). The Flemish version of the scale has been validated in a non-clinical sample and is used in this study (Engelen et al., 2006). For positive affect, Cronbach’s α was .87 at baseline and .88 at post-measurement. For negative affect, Cronbach’s α was .83 and .86 at baseline and post-measurement, respectively.

*Peace, meaning in life, and faith*. These three outcomes were assessed with the Dutch version of the Functional Assessment of Chronic Illness Therapy—Spiritual Well-Being scale (FACIT-Sp-12) (Cella et al., 1993; Peterman et al., 2002). Peace is about the experience of inner harmony, inner comfort, and peace of mind (e.g., “I feel a sense of harmony within myself”). Meaning in life refers to the view on purpose, on meaning in life, and on the reason(s) for living (e.g., “My life has been productive”). The scale on faith includes items about faith/spiritual beliefs and the role of faith/spiritual beliefs during illness (e.g., “I find strength in my faith or spiritual beliefs”). Each scale comprises four items. Answers were rated on a 5-point Likert scale from *not at all* to *very much*. At baseline, peace, meaning in life, and faith have Cronbach’s α of .78, .80, and .74, respectively. At post-measurement, Cronbach’s α was, respectively, .79, .82, and .78.

*Scottish PROM*. The Scottish Patient Reported Outcome Measure (PROM) assesses the outcomes of chaplaincy interventions (Snowden & Telfer, 2017). It comprises five items, namely being honest toward oneself, being anxious (reversed), having a positive outlook on life, feeling in control of life, and feeling a sense of peace. All the items were rated on a 5-point Likert scale from *never* to *always*. The Dutch translation was obtained via the European Research Institute for Chaplains in Healthcare (ERICH). In this study, the Cronbach’s α was .65 at baseline and .69 at post-measurement.

*Sociodemographic and religious/spiritual background variables*. Age, gender, nationality, civil status, educational level, and length of stay in the hospital were assessed. The religious/spiritual background of participants was measured with five single items, as shown in Table 1. First, on a 4-point Likert scale, the importance of religion/spirituality in patients’ life was assessed. Second, to get more insight into geriatric patients’ religious/spiritual profile, they had to describe whether they are non-believers, believers feeling affiliated to a religious tradition, or believers without any religious affiliation. Third, if applicable, they were asked which religion or other spiritual tradition they affiliate with. Finally, patients were asked how frequently they are involved in praying (alone or with other people) and in religious activities (in group, such as church activities). At both measurements, patients were asked whether or not they have talked to someone other than the chaplain about their doubts, worries, or fears, and, if applicable, with whom.

## Data Analysis Plan

All analyses were carried out by use of SPSS 27. Prior to the main analyses, data were screened for missing answers, drop-outs, and for potential nesting within the chaplain and hospital. First, we observed that most participants completed the whole questionnaire. Only a few missing answers were detected in the outcome variables (range for each item = 0–4). Second, reasons for drop-outs (see Fig. 1) and differences in study variables between those who dropped out (*n* = 88) and those who participated in the full study (*n* = 328) were analyzed. Sociodemographic and religious background variables and outcome scores were not related to whether or not study participants dropped out. A significant difference between the two groups was found only for the hospital setting in which they stayed; χ^2^(2) = 7.776, *p* = .020. Third, we checked for potential nesting of the outcome scores of the baseline measurement within the chaplains and within the hospitals. Variance in the outcome scores within the chaplains was not significant (*p* > .05). Also within the hospitals, the variance in the outcome scores was not significant (*p* > .05) or not found (intraclass correlation coefficient = 0).

To examine whether the chaplain’s intervention has an effect on the outcomes of the intervention group compared to the comparison group, while taking into account the groups' baseline scores, multilevel modeling (MLM) was used (Heck et al., 2022). MLM was performed by use of the MIXED procedure, with each outcome tested separately. Missing data and drop-outs were imputed by maximum likelihood estimation. This way of imputing missing data “uses all of the available data (complete and incomplete) to identify the parameter values that have the highest probability of producing the sample data” (Baraldi & Enders, 2010, p. 18). Age, gender, educational level, civil status, length of stay in the hospital, and the religious background variables were added as control predictors. Also, we controlled for the fact that some people have talked to someone other than the chaplain about their doubts, worries, or fears between the two measurements. Nationality was not included in the model as a control predictor, due to low variance in responses. All the variables we controlled for in the analyses are called “background variables”.

A high correlation was detected between the items measuring how often participants pray (“PRAY”), how often they are involved in religious activities (“RACT”), and how important religion/spirituality is in their life (“IMPT”); PRAYxRACT, *r* = .727; PRAYxIMPT, *r* = .773; RACTxIMPT, *r* = 752, all *p* < .001. Therefore, these items were merged into one variable “praying/religious activity/importance”. As the outcomes were not clustered in the hospitals or within the chaplains, we did not include the hospitals and chaplains as random factors into the multilevel model. Only the participant was included in the model as a random intercept to account for the variance within the geriatric patient. The group to which a participant was assigned (“group”), the time of the measurements (“time”), and the interaction between group and measurement time (“group-by-time”), were put into the model as fixed factors.

To adjust for multiple testing and to decide whether results are significant, we applied the Benjamini–Hochberg procedure to control false discovery rate (Benjamini & Hochberg, 1995). We took into account that no more than 5% of the significant tests can be false positives.

## Results

### Preliminary Analyses

No significant differences were identified between the intervention and comparison groups at baseline concerning age, gender, educational level, civil status, length of stay in the hospital, importance of religion/spirituality in life, religious affiliation, religious identification, and frequency of praying. Regarding involvement in religious activity, participants in the intervention group were significantly more involved in religious activities (*M* = 2.60, *SD* = 1.29, range = 1–4), compared to the participants in the comparison group (*M* = 2.30, *SD* = 1.24, range = 1–4); *t*(414) = − 2.32, *p* = .02.

According to the outcome variables, the levels of state anxiety and negative affect at baseline were significantly higher in the intervention group, compared to the comparison group. The baseline scores for hope, positive affect, peace, meaning in life, and the Scottish PROM were significantly higher in the comparison group, compared to the intervention group. Only faith showed no significant differences between the two groups at baseline. Means and standard deviations of the outcomes in both groups at baseline and the differences between the outcomes of both groups at baseline are displayed in Table 2.

**Table 2:** Means and standard deviations of the outcomes in the intervention and comparison group at baseline and the differences between the outcomes of both groups at baseline

Outcome measure	Intervention group		Comparison group		*t*	*df*	*p*	Range of the scale
	*M*	*SD*	*M*	*SD*				
State Anxiety	14.38	4.56	11.63	3.98	− 6.16	270.57	< .001	6–24
Hope	30.88	5.86	33.53	5.35	4.68	414	< .001	12–48
Positive affect	23.55	8.02	27.75	8.79	4.82	414	< .001	10–50
Negative affect	20.47	8.80	15.33	6.68	− 6.19	241.79	< .001	10–50
Peace	7.29	4.25	9.85	3.84	6.25	414	< .001	0–16
Meaning in life	10.71	4.09	11.98	3.64	3.14	274.98	< .001	0–16
Faith	5.92	4.46	5.74	4.00	− .43	414	.67	0–16
Scottish PROM	12.01	4.03	13.96	3.83	4.88	414	< .001	0–20

Means were calculated if all items were completed

### Outcome Analyses

**Fig. 2:**
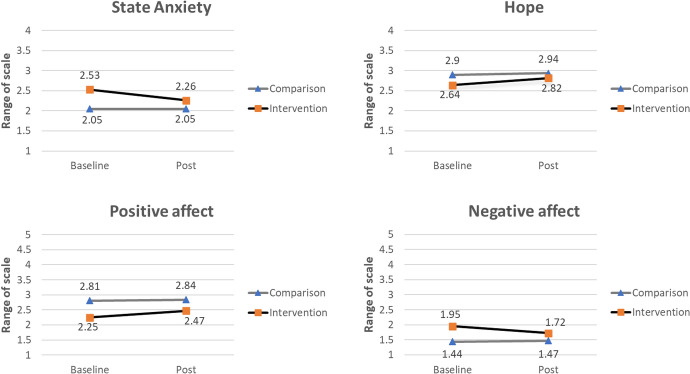
Estimated marginal means and interaction effects between group and time of state anxiety, hope, positive affect, and negative affect in the intervention and comparison group, at baseline and post-measurement, adjusted for background variables. *Note* A significant group-by-time interaction effect was found for state anxiety, state hope, positive affect, and negative affect. The standardized errors for these means are between .08 and .16

**Fig. 3:**
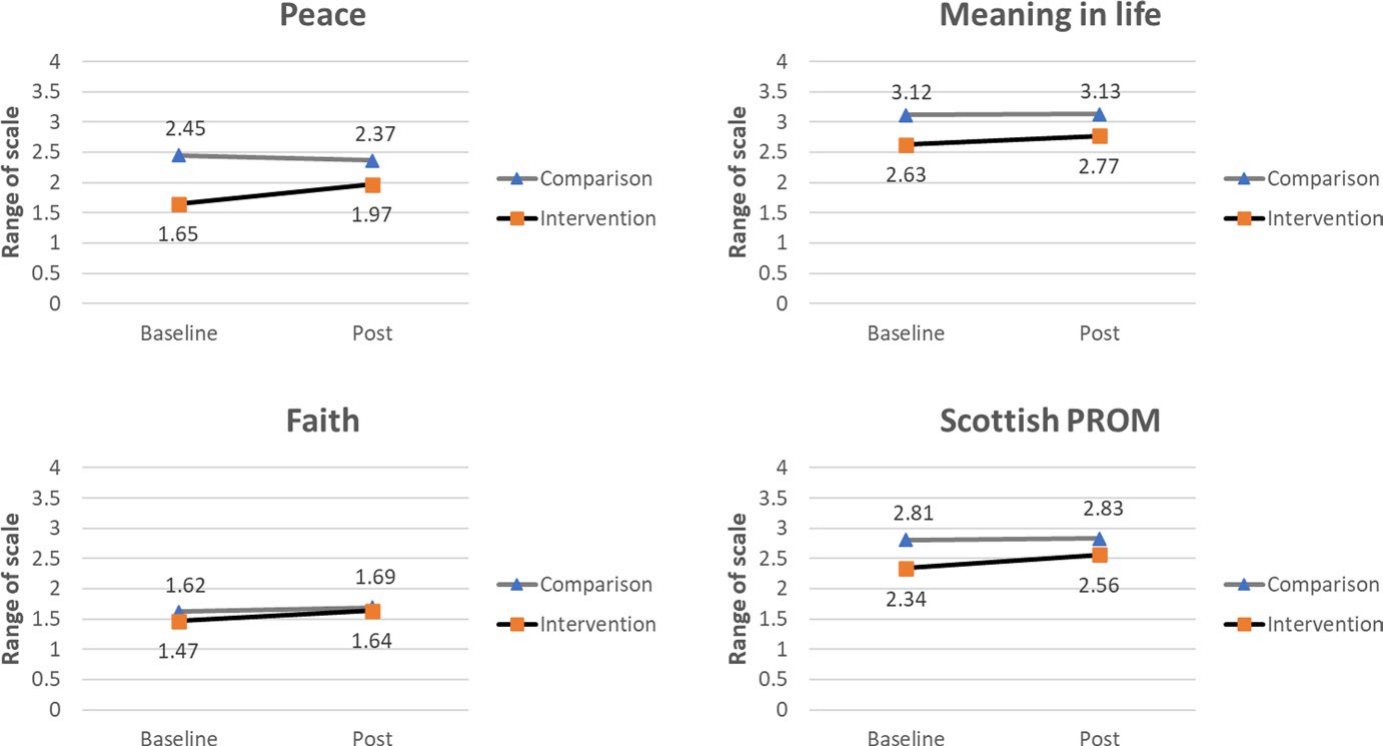
Estimated marginal means and interaction effects between group and time of peace, meaning in life, faith, and Scottish PROM in the intervention and comparison group, at baseline and post-measurement, adjusted for background variables*. Note* A significant group-by-time interaction effect was found for peace and Scottish PROM-scores, but not for meaning in life and faith. The standardized errors for these means are between .12 and .18

Analyses showed a significant interaction effect between group and time for state anxiety, hope, positive affect, negative affect, peace, and the Scottish PROMs. For meaning in life and faith, no significant interaction effect between group and time was found. Each outcome analysis accounted for the baseline scores and background variables of the intervention and comparison group. Figures 2 and 3 show the estimated marginal means for the intervention and comparison group before and after the intervention, and the interaction effects between group and time. Table 3 provides a summary of the main and interaction effects for each outcome variable. After controlling for multiple testing, the effects remain significant and are, in general, not the result of testing many hypotheses. For each outcome measure, the Benjamini–Hochberg critical value is reported in the note to Table 3.

First, the intervention group showed a significant diminished level of state anxiety after the intervention, while the levels of state anxiety remained the same in the comparison group before and after the intervention (Fig. 2); *b* =  − 0.274, *t*(328.000) =  − 3.283, *p* = .001. None of the controlling variables were significant predictors in this model.

Second, the experience of hope significantly increased in the intervention group after the chaplaincy intervention, compared to the comparison group (Fig. 2); *b* = 0.143, *t*(321.807) = 3.379, *p* < .001. Civil status, age, and the combined variable “praying/religious activity/importance” were significant predicting variables for hope.

Third, positive affect significantly increased in the intervention group at post-measurement, compared to the comparison group (Fig. 2); *b* = 0.191, *t*(326.706) = 2.394, *p* = .017. Age appeared to be a significant predictor for positive affect, together with the combined variable “praying/religious activity/importance”. Negative affect declined in the intervention group at post-measurement, while it increased in the comparison group (Fig. 2); *b* =  − 0.269, *t*(328.000) =  − 3.431, *p* < .001. Gender was a significant predictor for negative affect.

Fourth, peace significantly increased in the intervention group after the intervention, while there was a decline in peace in the comparison group (Fig. 3); *b* = 0.396, *t*(326.131) = 3.775, *p* < .001. The combined variable “praying/religious activity/importance” appeared to be a significant predictor for peace.

Finally, a significant interaction effect between group and time was found for the scores on the Scottish PROM-questionnaire. Scottish PROM-scores significantly increased in the intervention group after the intervention of the chaplain, compared to scores of the comparison group (Fig. 3); *b* = 0.207, *t*(323.242) = 2.621, *p* = .009. Again, “praying/religious activity/importance” was a significant predictor.

For meaning in life and faith, the data showed no significant group-by-time interaction effect; respectively *b* = 0.126, *t*(326.143) = 1.524, *p* = .128 and *b* = 0.096, *t*(325.567) = 1.270, *p* = .205. What the data did show was a significant effect of group on meaning in life and an effect of time on faith (Table 3). The level of meaning in life was indeed higher in the comparison group, compared to the intervention group across measurement time points. Faith increased from baseline to post-measurement across groups (Fig. 3). For both outcomes, “praying/religious activity/importance” was a significant predictor. For meaning in life, civil status was also a significant predictor. For faith, age was also a significant predictor.

**Table 3:** Main effects and interaction effects on state anxiety, hope, positive affect, negative affect, peace, meaning in life, faith, and Scottish PROM

	State anxiety			Hope			Positive affect			Negative affect		
Effect	*df*	*F*	*p*	*df*	*F*	*p*	*df*	*F*	*p*	*df*	*F*	*p*
Intercept	1, 328	283.069	< .001	1, 325	671.848	< .001	1, 327	159.951	< .001	1, 328	120.678	< .001
Gender	1, 328	1.487	.224	1, 326	0.014	.905	1, 327	0.235	.628	1, 328	4.212	.041
Educational level	3, 328	1.819	.143	3, 327	0.242	.867	3, 327	0.508	.677	3, 328	0.261	.854
Civil status	4, 328	0.357	.839	4, 325	3.894	.004	4, 327	2.086	.082	4, 328	0.372	.828
Length of stay	6, 328	0.686	.661	6, 325	0.738	.620	6, 327	0.579	.747	6, 328	0.733	.624
Intervention by others	1, 328	3.471	.063	1, 326	0.441	.507	1, 327	0.486	.486	1, 328	1.422	.234
Religious affiliation	2, 328	1.117	.329	2, 324	1.302	.273	2, 327	0.751	.473	2, 328	0.765	.466
Religious identification	2, 328	0.739	.478	2, 324	2.185	.114	2, 327	0.158	.854	2, 328	1.508	.223
Praying/religious activity/importance	1, 328	3.096	.079	1, 326	28.276	< .001	1, 328	7.821	.005	1, 328	0.924	.337
Age	1, 328	0.332	.565	1, 325	4.312	.039	1, 327	5.518	.019	1, 328	0.359	.549
Group	1, 328	24.518	< .001	1, 326	17.522	< .001	1, 327	28.911	< .001	1, 328	23.963	< .001
Time	1, 328	10.382	.001	1, 322	27.324	< .001	1, 327	9.703	.002	1, 328	6.752	.010
Group*Time	1, 328	10.779	.001	1, 322	11.420	< .001	1, 327	5.731	.017	1, 328	11.775	< .001

	Peace			Meaning in life			Faith			Scottish PROM		
Effect	*df*	*F*	*p*	*df*	*F*	*p*	*df*	*F*	*p*	*df*	*F*	*p*
Intercept	1, 328	69.663	< .001	1, 329	150.558	< .001	1, 326	2.245	.135	1, 325	255.518	< .001
Gender	1, 327	2.023	.156	1, 328	0.977	.324	1, 326	0.255	.614	1, 325	3.435	.065
Educational level	3, 327	0.931	.426	3, 328	0.248	.863	3, 326	0.535	.658	3, 325	0.781	.505
Civil status	4, 327	0.380	.823	4, 327	2.920	.021	4, 326	1.586	.178	4, 325	1.038	.388
Length of stay	6, 328	1.339	.239	6, 328	0.689	.659	6, 326	0.617	.717	6, 325	0.864	.522
Intervention by others	1, 327	0.012	.913	1, 328	0.636	.426	1, 326	0.088	.766	1, 325	1.309	.253
Religious affiliation	2, 327	1.457	.234	2, 327	0.701	.497	2, 326	1.020	.362	2, 324	2.512	.083
Religious identification	2, 327	0.623	.537	2, 327	0.930	.396	2, 326	3.010	.051	2, 324	2.056	.130
Praying/religious activity/importance	1, 330	8.014	.005	1, 329	11.934	< .001	1, 328	326.874	< .001	1, 327	5.061	.025
Age	1, 327	1.561	.212	1, 327	1.862	.173	1, 326	9.275	.003	1, 325	0.212	.646
Group	1, 327	36.274	< .001	1, 327	19.456	< .001	1, 326	1.846	.175	1, 325	22.752	< .001
Time	1, 326	5.448	.020	1, 326	3.627	.058	1, 326	9.999	.002	1, 323	8.708	.003
Group*Time	1, 326	14.251	< .001	1, 326	2.324	.128	1, 326	1.613	.205	1, 323	6.867	.009

The Benjamini–Hochberg critical-values are .006 for hope, .013 for negative affect, .019 for peace, .025 for state anxiety, .031 for the Scottish PROM, .038 for positive affect, .044 for meaning in life, and .05 for faith

## Discussion

The quasi-experimental study demonstrated a significant change in the intervention group on geriatric patients’ feelings of state anxiety, experience of hope, positive and negative affect, peace, and on the Scottish PROM-scores, compared to the comparison group. After the chaplaincy intervention, the levels of state anxiety and negative affect significantly decreased in the intervention group, compared to the group who received no intervention by the chaplain, while taking into account the background variables and the baseline scores of both groups. The levels of hope, positive affect, peace, and Scottish PROM-scores significantly increased in the intervention group, compared to the group who received no intervention by the chaplain, while taking into account the background variables and the baseline scores of both groups. For meaning in life and faith, no significant effect was found. Below, we describe whether these results are in line with other research, how the results could be explained, and what this means for further outcome research in chaplaincy care.

The significant changes in outcomes found in this study are in line with previous studies on chaplaincy care. Hope, peace, anxiety, and positive/negative affect are indeed aspects the chaplain cares for and reasons to refer to the chaplain. First, hope and peace have been pointed out by chaplains as themes that often occur during their interventions (Kwak et al., 2022). Chaplains care for patients’ hope and peace by being a witness of hope, by identifying and nurturing patients’ hope and inner peace resources, and by sharing and fostering a sense of hope and peace in patients (Massey et al., 2015; Nolan, 2011; VandeCreek & Lucas, 2001). Second, the care for anxiety is part of chaplaincy care. Patients appreciate the visit of a chaplain when they feel anxious (Piderman et al., 2010). Moreover, it is a frequently cited reason why caregivers refer to the chaplain (Chapman & Grossoehme, 2002; Flannelly et al., 2003; Fogg et al., 2004). Third, this also applies to positive and negative affect. The study by Galek and colleagues (2009) found that professional caregivers referred to the chaplain when negative affect was detected in the patient, especially grief, sadness, and anxiety. This was different when patients themselves requested a chaplaincy visit, or when family or volunteers referred to the chaplain. In these (self-)referrals, the patient experienced mainly positive affect, especially gratitude. These results suggest that referrals by professional caregivers are based mainly on patients’ negative affect, which was not the case for referrals made by the patient, family, and volunteers.

Also, the significant change found on the Scottish PROM-scores in this study is consistent with findings in previous research. As described at the beginning of this paper, two other studies showed an increase in the PROM-scores after patients received spiritual care (Snowden et al., 2022a; Tan et al., 2020). These studies either did not include a comparison group, or they focused on spiritual care provided by the whole healthcare team. Our study included a comparison group and focused on spiritual care provided by the chaplain. In this way, this study contributes to the further validation of the PROM-questionnaire in chaplaincy care. However, due to the variety of items in the PROM-questionnaire, it is difficult to clarify on what outcomes the chaplain’s intervention exactly has an impact. Together with the shortness of the scale, the instrument may therefore be more suitable as a screening instrument (Snowden et al., 2022b). In further analyses, it would be interesting to consider the items separately.

Next to the significant changes in outcomes in this study, there were two outcome measures that did not show a significant change, specifically meaning in life and faith. These scales refer to the experiences of the patients that their life is meaningful and that they find support in their faith. These findings are partly in line with a pilot study examining the outcomes of chaplaincy care in patients with advanced cancer (Kestenbaum et al., 2017). After the patients received three interventions by the chaplain, the study showed a significant increase in faith but not in meaning in life. That neither meaning in life nor faith changed significantly after the intervention of the chaplain in this study is difficult to explain. However, two possible factors can partly contribute to an explanation.

First, it may be an indication that perspectives on meaning in life and faith do not simply change after one encounter with the chaplain. Crystal Park and Susan Folkman (1997) consider people’s sense of meaning in life and their religion/spirituality as part of people’s global meaning framework. This framework provides meaning to situations and experiences and structures people’s life (Park, 2005, 2010). Based on Park’s robust global meaning framework, it is unlikely that what gives meaning and structure to life and life experiences change in a few days or after one intervention by the chaplain. This in contrast to outcomes such as feeling anxious or having a positive or negative affect, which may have the potential to evolve and change throughout the day or across two days. That does not mean that themes such as meaning in life and faith cannot be addressed during chaplaincy interventions. For some patients, the religious/spiritual identity and religious/spiritual support of the chaplain are highly valued (King, 2012). In fact, the aim of the chaplain is to support and facilitate patients’ reflection on meaning in life and their contemplation on the role of religion/spirituality in life.

Second, the way meaning in life and faith are measured by the FACIT-Sp-12 has been reviewed as problematic in earlier studies (Damen et al., 2022; Monod et al., 2015). As the subscale on meaning in life does not make a distinction between meaning in life and purpose, it is not adapted to the experiences of (older) people (Monod et al., 2015). Having no purpose in old age is not the same as experiencing a lack of meaning in life. For example, an older patient may no longer have purpose for the future but can still experience meaning in life based on great satisfaction with the purposes achieved in his/her past life (Monod et al., 2015). Regarding the faith subscale, three of the four items are not appropriate for patients who do not integrate religion or spirituality into their daily lives or during illness. For most patients who do not feel religiously/spiritually affiliated, the answer to these items remained “not at all”, both before and after the intervention (Damen et al., 2022).

When interpreting the results, we have to take four things into account. First, the differences in baseline scores between the intervention and comparison group. The fact that both groups had significantly different outcome scores at baseline makes the comparison between both groups more difficult. Although the analyses do account for the baseline scores, along with many background variables, we do have to interpret the results with caution. Second, the quasi-experimental design of the study needs to be taken into account. The two groups do not differ according to their sociodemographic background, but they do differ according to their perceived needs for chaplaincy care. As a result, we do not know what the effect of the intervention would be if both groups included people with and without a need for chaplaincy care. Third, the non-standardized referral needs some attention. Patients were referred to the chaplain based on the referrer’s personal assessment of patients’ need to receive chaplaincy care or not. The reason for non-standardized referrals is that we wanted to research a real-life setting in favor of the ecological validity of the study. At the same time, this limits the internal validity of the study. Moreover, the choice of non-standardized referrals has the consequence that it remains unclear what spiritual needs provoked a referral to the chaplain. Fourth, we have to take into account that the patients in the comparison group did not receive an alternative intervention. This means that we can describe the impact of chaplaincy as such, but we cannot compare it with the possible impact of spiritual care interventions by others. It would be interesting to include a comparison group in which patients receive spiritual care from a nurse, physician or volunteer. Although this would improve the study design, it does not reflect the real care context. In fact, in daily practice in Belgian hospitals, professional spiritual care is usually provided by the chaplain.

Nonetheless, this study does provide indications that healthcare chaplaincy is impactful for geriatric patients who are referred to the chaplain. This implies that the referrals made by the healthcare team are crucial. Therefore, screening and assessment tools to identify spiritual needs and training for caregivers on when to refer to a chaplain are needed to ensure the best possible (chaplaincy) care (Puchalski et al., 2006). Several assessment tools for spiritual needs have been developed that can help caregivers assess whether or not a referral to the chaplain is needed (Büssing, 2021; Monod et al., 2012).

This study contributes to the need expressed by chaplains to articulate the impact of their care more profoundly (Damen et al., 2020a; Handzo et al., 2014). However, since outcome studies in chaplaincy research have been published, chaplains and researchers formulated their hesitations toward this kind of research. They wonder if outcome research is able to capture the richness and specificity of healthcare chaplaincy (Damen et al., 2020b). Moreover, they indicate that outcome research tends to overlook key elements of healthcare chaplaincy. For example, it has been criticized that most outcome research does not take into account the importance of the trusting relationship between the chaplain and the patient in combination with the role of the personality of the chaplain (Nolan, 2015). To conduct proper outcome research in healthcare chaplaincy, these considerations need to be taken into account alongside a clearly defined goal of outcome research. In fact, outcome research does not aim to capture the uniqueness of chaplaincy care, but rather to get more insight into how chaplaincy interventions can contribute to patients’ well-being and to healthcare. When considering the results of outcome research, and of this study, we have to take two things into account. First, the results of outcome studies need to be interpreted together with other evidence-based research such as research on the processes/interventions that take place during chaplaincy interventions and qualitative research such as case studies. Second, we need to be aware of the intangibility of the transcendent nature of a chaplain-patient encounter. In doing so, we believe that outcome research can simultaneously respect the core of healthcare chaplaincy and articulate the impact of chaplaincy care.

## Limitations and Further Research

This study is affected by five limitations.

First, the intervention and comparison group were not randomized. They were composed based on caregivers’/interviewers’ estimation of patients’ need to receive or not receive chaplaincy care, and the baseline scores of the two groups were significantly different from each other. In further research, an RCT is recommended in which the two groups are completely randomized. However, conducting an RCT is often unfeasible or unethical in an acute healthcare context. In this study, we agreed that it would be unethical to deny chaplaincy care to patients who need it. Moreover, we wanted to investigate the impact of healthcare chaplaincy in the real hospital setting, where chaplains mostly visit those with spiritual needs and/or a need for chaplaincy care.

Second, chaplains’ interventions were not standardized. Since chaplains tailor their intervention to the patients’ occurring spiritual needs, measuring a standardized intervention would not accurately reflect the real care by the chaplain. Further research could test whether standardized chaplaincy interventions contribute to particular outcomes in geriatric care. For example, it would be interesting to know the effects of healthcare chaplaincy that focusses explicitly on the role of spirituality in geriatric patients’ life, or on the meaning and value of geriatric patients’ life story/ past life.

Third, we did not conduct a follow-up measure. In subsequent studies, it could be of interest to investigate long-term effects, especially in residential care for older adults. Recent studies in Belgian and Dutch hospitals have, so far, found no effects of spiritual/chaplaincy interventions after a couple of weeks or a couple of months (Buelens et al., 2023; Kruizinga et al., 2019).

Fourth, this study focused on the current population in geriatric wards who could speak Dutch. Further research could explicitly focus on patients with other languages, as well as other religious and cultural backgrounds. Given the pluralized context in West-Europe, further research could provide insight into how hospital chaplaincy can be optimized for geriatric patients with various (religious) beliefs/spiritual frameworks, cultures, and nationalities.

Last, patients’ answers might be affected by social desirability, as the interviews were conducted orally. Nevertheless, this was the best possible way to conduct the research, because it would be too difficult for the target group to complete the questionnaire alone. Also, the role of the interviewer in referring patients to the chaplain must be taken into account when interpreting the results. When the interviewer identified a need for chaplaincy care during baseline measurement, the interviewer decided to refer the patient to the chaplain. In the intervention group, 51.7% of the referrals were made by the interviewers. The high number of referrals by the interviewers can be explained by the fact that questions about the outcome measures (during baseline measurement) explicitly revealed the presence of spiritual needs. Since the interviewers are not part of the real care context, this limits the (generally high) ecological validity of the study.

## Conclusion

The study is rather unique in showing that healthcare chaplaincy has an effect on aspects of geriatric patients’ well-being and distress. The value of this study lies also in the fact that the results of this study are based on older patients’ own experiences, which are often unheard in research. Since our findings show that geriatric patients can benefit from chaplaincy interventions, it might be good to consider how chaplaincy care can be integrated into the care for older adults. For chaplains, the study provides concrete data and an evidence-based language about the outcomes of their interventions, which can help them in the communication with other caregivers about their work. It can encourage chaplains to reflect on their own work not only in terms of concrete tasks and aims but also very specifically in terms of outcomes. Through this study, we hope that other researchers feel encouraged to conduct research on the outcomes of chaplaincy care for older adults, in conjunction with studies on what chaplains do as well as with studies on older patients’ lived experience with chaplaincy care.

## Data Availability

The data presented in this study are available on request from the corresponding author.
